# Systematic review of pediatric health outcomes associated with childhood adversity

**DOI:** 10.1186/s12887-018-1037-7

**Published:** 2018-02-23

**Authors:** Debora Lee Oh, Petra Jerman, Sara Silvério Marques, Kadiatou Koita, Sukhdip Kaur Purewal Boparai, Nadine Burke Harris, Monica Bucci

**Affiliations:** 1 Center for Youth Wellness, 3450 Third Street, Bldg 2, Ste 201, San Francisco, CA 94124 USA; 2 Human Impact Partners, Oakland, California, USA

**Keywords:** Systematic review, Childhood adversity, Pediatric health outcomes, Toxic stress, Adverse childhood experiences

## Abstract

**Background:**

Early detection of and intervention in childhood adversity has powerful potential to improve the health and well-being of children. A systematic review was conducted to better understand the pediatric health outcomes associated with childhood adversity.

**Methods:**

PubMed, PsycArticles, and CINAHL were searched for relevant articles. Longitudinal studies examining various adverse childhood experiences and biological health outcomes occurring prior to age 20 were selected. Mental and behavioral health outcomes were excluded, as were physical health outcomes that were a direct result of adversity (i.e. abusive head trauma). Data were extracted and risk of bias was assessed by 2 independent reviewers.

**Results:**

After identifying 15940 records, 35 studies were included in this review. Selected studies indicated that exposure to childhood adversity was associated with delays in cognitive development, asthma, infection, somatic complaints, and sleep disruption. Studies on household dysfunction reported an effect on weight during early childhood, and studies on maltreatment reported an effect on weight during adolescence. Maternal mental health issues were associated with elevated cortisol levels, and maltreatment was associated with blunted cortisol levels in childhood. Furthermore, exposure to childhood adversity was associated with alterations of immune and inflammatory response and stress-related accelerated telomere erosion.

**Conclusion:**

Childhood adversity affects brain development and multiple body systems, and the physiologic manifestations can be detectable in childhood. A history of childhood adversity should be considered in the differential diagnosis of developmental delay, asthma, recurrent infections requiring hospitalization, somatic complaints, and sleep disruption. The variability in children’s response to adversity suggests complex underlying mechanisms and poses a challenge in the development of uniform diagnostic guidelines. More large longitudinal studies are needed to better understand how adversity, its timing and severity, and the presence of individual genetic, epigenetic, and protective factors affects children’s health and development.

## Background

Evidence has shown that adversities such as abuse or neglect experienced during childhood can have lifelong consequences by affecting the foundations of health [1–4]. This body of evidence has generated growing attention to the concept of *toxic stress*, the chronic or frequent activation of the stress response from exposure to serious childhood adversity in the absence of adequate support or protection from adults [5]. Through a mechanism that is influenced by genetic, social, and biological factors, exposure to childhood adversity has been linked to the dysregulation of the neuroendocrine immune circuitry, which results in alterations of brain architecture and other organ systems during sensitive periods of development [6, 7]. Negative health outcomes that follow later in life reflect the physiological, epigenetic, and cognitive consequences that the brain and body pay for adapting to stressful and traumatic experiences [2, 8].

In one of the earliest studies examining the association between cumulative childhood adversity and health outcomes, known as the Adverse Childhood Experiences (ACE) Study, Felitti et al [9] found a dose-response relationship between ACEs and many leading causes of death in adults, such as chronic respiratory disease, stroke, heart disease, cancer, and diabetes. A growing body of research has since further confirmed that childhood adversity is associated with chronic disease and early death [10–15]. Hence, early detection and intervention can have a positive, lifelong impact on an individual’s health and well-being. Current efforts to address toxic stress in children are limited by gaps in understanding of the biological response to childhood adversity, and much of the existing evidence is based on adult studies with retrospective reports of adversity in childhood [15–17]. Studies on childhood adversity with pediatric health outcomes have pointed to an increased likelihood of physical and developmental health issues in children exposed to adversity [18–20], but these studies have been primarily cross-sectional, making it difficult to determine the temporal relationship between exposure and outcome. The present systematic review, therefore, was focused on longitudinal studies in children to better understand the biological mechanisms linking exposure to childhood adversity with pediatric health outcomes.

## Methods

We followed the PRISMA Statement [21] in the conduct and reporting of this systematic review.

### Data sources

PubMed, PsycArticles, and CINAHL were searched for the following terms in the title or abstract: (“early life” OR adolescent OR child OR infant OR youth OR childhood OR prenatal OR “in utero”) AND (divorce OR “parental incarceration” or “parental depression” OR abuse OR neglect OR adversity OR maltreatment OR “toxic stress” OR “allostatic load” OR “adverse childhood experience”). Other terms relevant to childhood adversity such as “trauma,” “parental mental health,” and “parental substance abuse” were not included as search terms because they were not specific enough to identify additional relevant articles. The searches were limited to full-text articles in the English language, published between January 1, 2001 and December 31, 2015. In addition, the searches excluded animal studies, case reports, review articles, and qualitative studies.

### Study selection

Title and abstracts were reviewed and nonrelevant records (studies with no exposure to childhood adversity and/or biological health outcomes) were excluded. Exposure to childhood adversity was defined as exposure to 1 or more of the experiences shown in Table 1, which were derived from the Center for Youth Wellness ACE Questionnaire (CYW ACE-Q) [22]. The CYW ACE-Q was designed to be administered to children aged 0–19 years and includes the traditional 10 ACEs from the ACE Study, as well as additional early life stressors identified by experts and community stakeholders. An adverse exposure in-utero (e.g. maternal drug use) was part of the original search criteria, but these studies were subsequently excluded due to conceptual differences in exposures and outcomes. Biological health outcomes were defined as objective and specific developmental or clinical changes in the health of children. Mental and behavioral health outcomes were excluded, as were direct and immediate physical effects of adverse incidents (i.e. abusive head trauma).

**Table 1:** Adverse Childhood Experiences (ACEs), age 0–19 years

Category	Definition
Abuse	Someone pushed, grabbed, slapped or threw something at child or child was hit so hard that she/he was injured or had marks
	Household member swore at, insulted, humiliated, or put down child in a way that scared child or household member acted in a way that made child afraid that she/he might be physically hurt
	Someone touched child’s private parts or asked child to touch that person’s private parts in a sexual way that was unwanted, against child’s will, or made child feel uncomfortable
Neglect	More than once, child went without food, clothing, a place to live, or had no one to protect her/him
	Child often felt unsupported, unloved, and/or unprotected
Household dysfunction	Child’s parents or guardians were separated or divorced
	Child saw or heard household members hurt or threaten to hurt each other
	Household member was depressed, mentally ill, or attempted suicide
	Household member had a problem with drinking or using drugs
	Household member served time in jail or prison
Other adversities	Child was separated from primary caregiver through deportation or immigration
	Child had a serious medical procedure or life threatening illness
	Child experienced harassment or bullying at school
	Child experienced verbal or physical abuse or threats from a romantic partner (i.e. boyfriend or girlfriend)
	Child often saw or heard violence in the neighborhood or school
	Child was detained, arrested, or incarcerated
	Child was often treated badly because of race, sexual orientation, place of birth, disability, or religion
	Child lived with a parent or guardian who died
	Child was in foster care

The categories and definitions were derived from Center for Youth Wellness ACE Questionnaire [22], which was adapted from the original ACE Study [9]

**Fig. 1:**
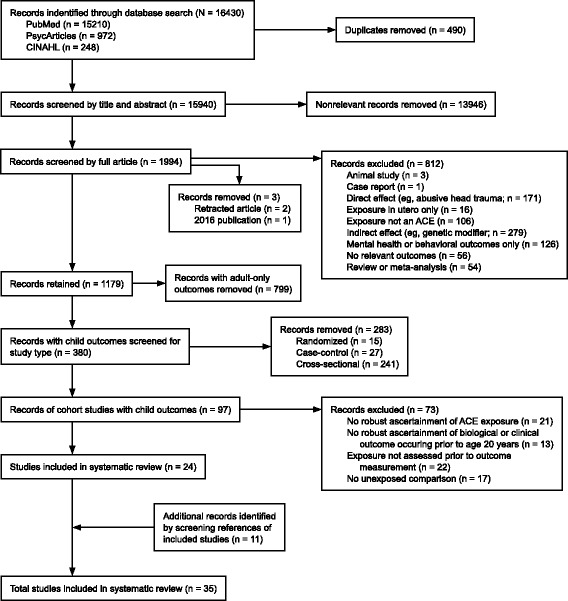
Flow diagram of studies included in the systematic review

The first two authors (D.O. and P.J.) evaluated the full text of the retained records using eligibility criteria shown in Fig. 1. Subsequently, only records of prospective and retrospective cohort studies examining child outcomes occurring prior to the age of 20 years were retained. The retained records were further evaluated using the following inclusion criteria: 1) robust ascertainment of an ACE exposure, as defined in Table 1; 2) robust ascertainment of a biological health outcome, excluding general measures of health, health-related quality of life, and death; 3) exposure measured prior to outcome; and 4) use of an unexposed comparison group. Each study was reviewed by the 2 authors, and disagreements were resolved through discussion. As a final step in study selection, the references of the selected studies were searched for additional studies that fit all inclusion criteria.

### Data extraction and synthesis

We extracted data on setting, study design, sample size, sample description, length of follow-up, adverse exposures, biological health outcomes, and relevant main findings (Tables 2–3). When both simple and adjusted analyses were reported, we extracted the findings that adjusted for covariates and/or confounders. The data were narratively synthesized and organized by type of biological outcome.

**Table 2:** Characteristics of included cohort studies examining the association between childhood adversity and child developmental outcomes

First author (year, setting)	Sample description	Exposures^a^	Outcomes	Findings (95% CI)
Boynton-Jarrett (2012, UK) [26]	Birth cohort, followed to age 42 years (*N* = 4524)	1 and 2 or more forms of childhood neglectful environment (physical neglect, maladjustment, mental subnormality in the family, bullying, contact with social services), assessed at age 7 years or at both ages 7 and 11 years	Onset of menarche assessed at age 16 years, categorized into early menarche (<= age 11 years), average menarche (age 12–13 years), and late menarche (=> age 14 years)	1 form of neglectful environment, OR = 1.21 (1.03–1.43), and 2 or more forms, OR = 1.76 (1.41–2.20), were both significantly associated with late menarche; neither 1 nor 2 or more forms were significantly associated with early menarche, OR = 0.90 (0.73–1.10) and OR = 1.21 (0.91–1.63), respectively
Enlow (2012, USA) [27]	Birth cohort, followed to age 5 years (*N* = 206)	Interpersonal trauma exposure (physical abuse, psychological maltreatment, neglect during age 0–24 month, sexual abuse during age 24–64 months, witnessing partner violence against the mother during age 0–24 or 24–64 months)	Cognitive functioning (mental and motor development, intelligence) from 24 months to 96 month of age	Interpersonal trauma exposure during age 0–24 months was significantly associated with cognitive scores, *P =* .002; there was a nonsignificant association for exposure during age 24–64 months, *P =* .84
Li (2004, UK) [24]	Birth cohort, followed to age 33 years (*N* = 7697)	Parental separation or divorce by age 7 years	Male height SD scores at ages 7, 11, and 16 years	Parental separation or divorce was associated with lower scores at ages 7, 11 (*p* < .05), and 16 years (*P =* ns)
		Parental separation or divorce by age 7 years	Female height SD scores at ages 7, 11, and 16 years	Parental separation or divorce was nonsignificantly associated with lower scores at ages 7, 11, and 16 years
Li (2004, UK) [25]	7993 birth cohort members, followed to age 33 years, and 2462 of their offspring	Parental separation or divorce by age 7 years	Height SD scores at age 7 years for cohort members and at age 4–18 years for offspring	Parental separation or divorce was nonsignificantly associated with lower scores for both cohort members and offspring
Richards (2004, UK) [29]	Birth cohort, followed to age 53 years (*N* = 1339)	Parental divorce or death by age 8 years	Cognitive ability at ages 8 and 15 years	Parental divorce or death was significantly associated with lower cognitive ability at ages 8, *P =* .05, and 15 years, *P =* .001
Strathearn (2001, Australia) [28]	Birth cohort (born at <1000 g), followed to age 4 years (*N* = 269)	Referred or substantiated maltreatment (physical, emotional, sexual abuse; neglect) occurring prior to cognitive assessment	Cognitive delay at age 4 years	Referrals and substantiated referrals were significantly associated with cognitive delay, *P* < .001 and *P =* .003, respectively

All studies were prospective cohort studies

95% CI, 95% confidence interval; *OR*, odds ratio; *SD*, standard deviation

^a^The nonexposed comparison represents the absence of the examined exposure

**Table 3:** Characteristics of included cohort studies examining the association between childhood adversity and child clinical outcomes

First Author (year, setting)	Sample description	Exposures^a^	Outcomes	Findings (95% CI)
Armitage (2009, USA) [56]	18 children aged 2 weeks, followed to age 6 months	Maternal depression, assessed at enrollment or within 2 weeks postpartum	Average sleep time in 24 hours, sleep distribution (nocturnal vs. day), nocturnal sleep latency, sleep efficiency (awakening from sleep), and number of daytime sleep episodes, assessed at 2 and 24 weeks	Maternal depression was not associated with significant differences in average sleep time at 2 or 24 weeks, *P <* .13, but was associated with significantly shorter nocturnal sleep, *P =* .007, more awakenings during the night, *P <* .0001, longer nocturnal sleep latency, *P <* .0005, lower sleep efficiency, *P <* .0009, and more daytime sleep episodes of a shorter average duration, *P <* .0001
Ashman (2002, USA) [33]	72 children aged 14 months, followed to age 7–8 years	Maternal depression (assessed retrospectively when child was aged 14 and 24 months, and 3.5, 4.5, 6.5, and 7–8 years)	Salivary cortisol levels at age 7–8 years	Maternal depression was nonsignificantly associated with salivary cortisol levels and diurnal rhythm of cortisol
Boynton-Jarrett (2010, USA) [46]	Birth cohort, followed to age 60 months (*N* = 1595)	Maternal intimate partner violence (IPV; physical, sexual, and restrictive abuse), categorized into early IPV (reported at baseline and/or the 12-month assessment only); late IPV (reported at the 36- and/or 60-month assessment only); or chronic IPV (reported both at baseline and/or the 12-month assessment and at the 36- and/or 60-month assessment)	Obesity status at age 60 months	Early and late maternal IPV were associated with a borderline, nonsignificant increase in risk for obesity, OR = 1.12 (0.67–1.85) and OR = 1.25 (0.88–1.77), respectively; maternal chronic IPV was associated with a significantly higher risk for obesity, OR = 1.80 (1.24–2.61); analyses stratified by sex indicated a significantly increased risk for obesity only among females exposed to maternal chronic IPV, adjusted OR = 2.21 (1.30–3.75), as compared with males, OR = 1.66 (0.94–2.93)
Caserta (2008, USA) [38]	169 children aged 5–10 years, followed for 3 years	Parental psychiatric symptoms (including depression, anxiety, and psychoticism), assessed at the beginning of each 1-year period	Number of illnesses and febrile illnesses in the 1 year following assessments 1, 3, and 5	Parental psychiatric symptoms were associated with a significantly increased rate of illness , RR = 1.40 (1.06–1.85), *P =* .02, as well as febrile illness, RR = 1.77 (1.00–3.13), *P =* .05
		Parental psychiatric symptoms in the previous month, ascertained at 7 assessments in 6-month intervals	Natural killer cell function at each of the 7 assessments	Parental psychiatric symptoms were associated with significantly enhanced natural killer cell function, *P <* .01
Copeland (2014, USA) [40]	3 cohorts of children aged 9, 11, and 13 years at risk for psychiatric problems, followed to age 21 years (*N* = 1420)	Bullying in the 3 months prior to each assessment between ages 9 and 16 years (up to 9 waves)	C-reactive protein (CRP) levels at each assessment between ages 9 and 16 years	Bullying was not significantly associated with CRP levels when adjusted for either CPR-related covariates, *P =* .22, or bullying-related covariates, *P =* .10
Dreger (2010, Canada) [30]	315 children with asthma and 188 children without asthma, followed to age 7–10 years	Maternal distress (at least 1 physician diagnosis of a depressive or anxiety disorder or at least 1 prescription for antidepressant, anxiolytic, or hypnotic medication), categorized into postnatal (first year only), late (2–7 years of age), or recurrent distress (first year and 2–4 and/or 5–7 years)	Serum cortisol levels at age 7–10 years	Postnatal and recurrent maternal distress were significantly associated with higher cortisol levels, *P =* .007 and .006, respectively, but late distress was not, *P =* .112; the interaction between asthma diagnosis and recurrent distress was significantly associated with lower cortisol levels, *P =* .012, in children with asthma, whereas postnatal and late distress were not, *P =* .666 and .345, respectively; among children without asthma, cortisol levels were 46%, 26%, and 11% higher in children exposed to postnatal distress, recurrent distress, and late distress, respectively, *P <* .05; among children with asthma, cortisol levels were 33% higher and 13% lower in children exposed to postnatal distress and recurrent distress, respectively, *P <* .05 (the predicted change for late distress was near 0%)
Essex (2002, USA) [31]	282 children aged 1 month, followed to age 4.5 years	Clinically significant maternal depression in infancy (ages 1, 4, and 12 months), at age 4.5 years, or in both periods	Salivary cortisol levels at age 4.5 years	Maternal depression in both periods was associated with marginally significant, higher cortisol levels, *P =* .09, in comparison with no exposure or exposure at age 4.5 years; the association for exposure in infancy was not significant
Flaherty (2009, USA) [53]	805 children aged 4 or 6 years from 5 study sites, followed to age 12 years	Number of adversities during the first 12 years of child's life: maltreatment (psychological maltreatment, physical abuse, sexual abuse, neglect) and household dysfunction (caregiver substance use/alcohol abuse, depressive symptoms, being treated violently; and criminal behavior in household), assessed at ages 4, 6, 8, and 12 years	Somatic complaints at age 12 years (headaches, nausea, dizziness, tiredness, eye problems, aches, skin problems, stomach problems, vomiting, nightmares, and constipation)	5 or more adversities were not significantly associated with somatic complaints reported by child, OR = 1.87 (0.65–5.35), *P =* .24, but were significantly associated with somatic complaints reported by caregiver, OR = 4.26 (1.17–15.5), *P =* .03, and by either child or caregiver, OR = 2.72 (1.37–5.42), *P =* .01; with the exception of 3/4 adversities reported by either child or caregiver, OR = 2.38 (1.02–5.00), *P =* .04, fewer than 5 or more adversities reported by any source were not significantly associated with somatic complaints
		Number of adversities during the first 6 years of child's life (assessed at ages 4 and 6 years) and during the second 6 years (assessed at ages 8 and 12 years): maltreatment (psychological maltreatment, physical abuse, sexual abuse, and neglect) and household dysfunction (caregiver substance use/alcohol abuse, depressive symptoms, being treated violently; and criminal behavior in household)	Somatic complaints at age 12 years (headaches, nausea, dizziness, tiredness, eye problems, aches, skin problems, stomach problems, vomiting, nightmares, and constipation)	Adversity, particularly in the second 6 years of life, was associated with an increased risks of somatic complaints, but with differential effects by number of adversities and source of reporting: 5 or more adversities during the first 6 years of life were not significantly associated with child's report of somatic complaints, but were significantly associated with caregiver's report, OR = 3.31 (1.08–10.1), *P =* .04, and with either child's or caregiver's report, OR = 2.48 (1.05–5.87), *P =* .04; 5 or more adversities during the second 6 years of life were significantly associated with caregiver's report, OR = 3.37 (1.14–10.0), *P =* .03, but not with child's report of somatic complaints, OR = 0.70 (0.14–3.54), *P =* .67, or with either child's or caregiver's report, OR = 1.90 (0.73–4.96), *P =* .19
Flaherty (2013, USA) [54]	933 children aged 4 or 6 years from 5 study sites, followed to age 14 years	Number of adversities during ages 0–6 years, 6–12 years, and 13–14 years: maltreatment (psychological maltreatment, physical abuse, sexual abuse, and neglect) and household dysfunction (caregiver’s substance use/alcohol abuse, depressive symptoms, being treated violently; and criminal behavior in household)	Somatic concerns at age 14 years (headaches, nausea, dizziness, tiredness, eye problems, aches, skin problems, stomach problems, vomiting, nightmares, and constipation)	Adversities across all ages had a graded relationship with somatic concerns: the ORs for somatic concerns were 4.19 (0.50–34.90), 8.91 (1.15–68.83), and 9.25 (1.25–68.23) for 1, 2, and =>3 adversities, respectively; for children experiencing adversities during ages 0–6 years, the ORs for somatic concerns were 1.90 (0.81–4.47), 1.29 (0.52–3.24), and 2.12 (0.90–5.00) for 1, 2, and =>3 adversities, respectively; for children experiencing adversities during ages 6–12 years, the ORs were 1.50 (0.74–3.02), 1. 46 (0.71–3.01), and 1.08 (0.51–2.31) for 1, 2, and =>3 adversities, respectively; for children experiencing adversities during ages 13–14 years, the ORs were 1.67 (0.92–3.03), 2.27 (1.13–4.59), and 3.47 (1.61–7.50) for 1, 2, and =>3 adversities, respectively
Frohlich (2011, Germany) [49]	95 children aged 7–15 years, followed for 24 months	Maternal depression, assessed at baseline	Success vs. failure in weight reduction between assessment at baseline and assessment at 24 months	Psychosocial variables (family adversity index, maternal depression, and maternal attachment style) as a group were significantly predictive of weight reduction, *P <* .031, and maternal depression was the best predictor, *P <* .003
		Maternal depression, assessed at baseline	Weight change (BMI) between assessment at 12 months and assessment at 24 months	Psychosocial variables (family adversity index, maternal depression, and maternal attachment style) as a group were significantly predictive of weight change, *P <* .027, but maternal depression was not the best predictor, *P =* ns
Hairston (2011, USA) [57]	Children of 83 mothers with a history of child abuse and PTSD, 38 mothers with a history of child abuse but no PTSD, and 63 mothers with no history of child abuse or PTSD, followed from either 14–28 weeks gestation or 6–8 weeks postpartum to age 18 months	Maternal PTSD, assessed at intake and at age 4 months	Infant sleep at age 4 months (number and duration of awakenings per night in the past week, wake after sleep onset)	Maternal PTSD had a borderline, nonsignificant association with wake after sleep onset, *P =* .081, and a nonsignificant association with number and duration of awakenings per night in the past week
Halligan (2004, UK) [32]	48 children of mothers with postnatal depression and 39 children of nondepressed mothers, followed from age 2 months to 13 years	Maternal postnatal depression, assessed at age 2 months	8:00 am salivary cortisol levels at age 13 years	Maternal postnatal depression was significantly associated with 8:00 am cortisol levels over and above other factors (mean cortisol, *P <* .05; cortisol variability, *P <* .01)
Kozyrskyj (2008, Canada) [52]	Birth cohort, followed to age 7 years (*N* = 13907)	Maternal distress (physician visits, hospitalizations, or prescriptions for depression or anxiety), categorized into postpartum, short-term (at least 1 episode during year 1 and years 1 to 5 of child’s life), or long-term distress (from child’s birth to age 7 years (at least 1 episode during year 1, years 1 to 5, and years 5 to 7)	Asthma at age 7 years (at least 2 physician visits for asthma, 1 asthma hospitalization, or 2 prescriptions for any asthma drug in the year after the child’s seventh birthday)	Postpartum distress and short-term distress were not associated with a significant increase in risk of asthma, OR = 1.05 (0.79–1.41) and OR = 1.00 (0.72–1.37), respectively, but long-term distress was, OR = 1.25 (1.01–1.55)
Lange (2011, Puerto Rico) [51]	339 sets of twins, followed from birth to age 3 years (*N* = 678)	Maternal depressive symptoms; paternal PTSD, antisocial behavior, and depression; parental depression(number of parents with depression), all within the first year of child's life	Asthma symptoms in the previous 4 weeks and asthma hospitalizations in the previous year, both assessed at age 1 year	Paternal PTSD, OR = 1.08 (1.03–1.14), *P =* .003, antisocial behavior, OR = 1.09 (1.04–1.15), *P <* .001, and depression, OR = 9.95 (1.38–71.59), *P =* .02, as well as maternal depressive symptoms, OR = 1.13 (1.02–1.25), *P =* .02, and parental depression, OR = 1.70 (1.14–2.53), *P =* .01, were all associated with a significant increase in risk of recent asthma symptoms; maternal depressive symptoms were associated with a borderline, nonsignificant increase in risk for asthma hospitalizations, OR = 1.14 (0.98–1.33), *P =* .09; no other exposure was significantly associated with hospitalizations
		Maternal depressive symptoms; paternal PTSD, antisocial behavior, and depression; parental depression (number of parents with depression), all within the first year of child's life	Unplanned clinic or emergency department visit for asthma, use of oral steroids for asthma, asthma hospitalizations, and asthma diagnosis in the previous year, all assessed at age 3 years	None of the exposures was significantly associated with unplanned clinic or emergency department visit; only maternal depressive symptoms were associated with a significant increase in risk of asthma diagnosis, OR = 1.13 (1.01–1.27), *P =* .03, and a borderline, nonsignificant increase in risk of asthma hospitalizations, OR = 1.16 (1.00–1.36), *P =* .05; paternal depression was associated with a borderline, nonsignificant increase in risk for use of oral steroids, OR = 3.03 (0.84–10.97), *P =* .09; parental depression was associated with a borderline, nonsignificant increase in risk of asthma hospitalizations, OR = 1.86 (0.98–3.56), *P =* .06
Lanier (2010, USA) [50]^b^	3845 maltreated children and 2417 nonmaltreated children (matched on birth year, region of residence) from families receiving public assistance, followed from birth to age 22 years	Substantiated or unsubstantiated maltreatment report (neglect, abuse) prior to age 12 years	First hospital treatment for asthma episode prior to age 18 years, but after maltreatment report	Maltreatment was associated with a significantly increased risk for first hospital treatment for asthma, HR = 1.73 (1.47–2.04), *P <* .001
		Substantiated or unsubstantiated maltreatment report (neglect, abuse) prior to age 12 years	First hospital treatment for a nonasthma cardio-respiratory disease episode (e.g. chronic pharyngitis) prior to age 18 years, but after maltreatment report	Maltreatment was associated with a significantly increased risk for first hospital treatment for nonasthma cardio-respiratory disease, HR = 2.07 (1.87–2.29), *P <* .001
		Substantiated or unsubstantiated maltreatment report (neglect, abuse) prior to age 12 years	First hospital treatment for an infection (e.g. mycosis) prior to age 18 years, but after maltreatment report	Maltreatment was associated with a significantly increased risk for first hospital treatment for an infection, HR = 2.09 (1.85–2.36), *P <* .001
Lynch (2015, USA) [41]	93 maltreated children aged 4 years from an urban area and 93 nonmaltreated children of the same age from families receiving public assistance (demographically matched by gender, ethnicity, number of adults in the home, and family history of public assistance), followed to age 9 years	Documented physical neglect, physical abuse, sexual abuse, or emotional maltreatment prior to age 4 years; living in neighborhood characterized by violent crime (rape, homicide, aggravated assault, and robbery) at age 4 years	Change in respiratory sinus arrhythmia (RSA) at age 9 years	Maltreatment status and neighborhood crime were not significantly associated with RSA, *P* = .66 and .20, respectively; the interaction between cognitive challenge, maltreatment, and neighborhood crime was significantly associated with change in RSA, *P* = .01; the interaction between cognitive challenge, GABRA6 genotype, maltreatment, and neighborhood crime was significantly associated with change in RSA, *P* = .003
Margolin (2010, USA) [55]	103 children aged 9–10 years, followed for 3 years	Parent-to-youth aggression, marital physical aggression, and community violence in the previous 12 months, assessed across 3 waves approximately 1 year apart	Somatic complaints assessed at wave 3	Parent-to-youth aggression was significantly associated with a slightly increased risk of experiencing somatic symptoms, RR = 1.03 (1.01–1.05), *P <* .05, whereas marital physical aggression and community violence were not, RR = 1.01 and 1.03 (0.99–1.03 and 0.99–1.08), respectively; analysis of cumulative violence exposure indicated that each unit increase on the cumulative violence exposure index (0–9) was associated with an increased risk of experiencing somatic symptoms, RR = 1.12 (1.003–1.24), *P <* .05
Morris (2015, UK) [45]	Birth cohort, followed to age 17 years (*N* = 7021)	Parental separation or death by age 4 years	BMI trajectory from age 4 to 17 years	Parental separation was associated with a BMI that was 1.1% (0.2–2.0) higher at age 4 years, but this diminished by age 9 years (1.1%, 0–2.2) and further by age 17 years (0.5%, -1.3 to 2.2); parental death was associated with lower BMI throughout childhood and adolescence (insufficient power to reliably determine associations)
Noll (2007, USA) [48]	84 sexually abused females aged 6–16 years and 89 nonabused females (matched on age, residing zip codes, race/ethnicity, predisclosure SES, family constellation, and other nonsexual trauma), followed for approximately 7 years	Substantiated sexual abuse within 6 months of participation	Obesity status in childhood/early adolescence (age 6–14 years) and in middle/late adolescence (age 15–19 years)	Substantiated sexual abuse was associated with a nonsignificant increase in risk for obesity during childhood/early adolescence, OR = 1.25 (0.05–3.00), *P =* .52, and middle/late adolescence, OR = 2.03 (0.54–4.60), *P =* .09
Ouellet-Morin (2011, UK) [35]	30 pairs of 5-year-old identical twins, followed to age 12 years (*N* = 60), with co-twin never having experienced bullying victimization	Bullying victimization experienced at least occasionally, assessed at ages 7, 10, and 12 years	Salivary cortisol levels at age 12 years	Bullying victimization was significantly associated with a blunted cortisol response after a stress test, *P =* .02
Ouellet-Morin (2011, UK) [36]	95 pairs of 5-year-old identical twins, followed to age 12 years (*N* = 190), with co-twin never having experienced childhood maltreatment or frequent bullying	Childhood maltreatment (assessed at ages 5, 7, 10, and 12 years) or frequent bullying victimization (assessed at ages 7, 10, and 12 years)	Salivary cortisol levels at age 12 years	Maltreatment or frequent bullying was significantly associated with a blunted cortisol response following a stress test, *P =* .005
Peckins (2015, USA) [34]	303 maltreated children aged 9–12 years and 151 nonmaltreated children of the same age and residing in the same 10 zip codes, followed for 4.5 years	New substantiated referral for any type of maltreatment (sexual abuse, physical abuse, emotional abuse, or neglect) in the month prior to recruitment	Cortisol reactivity patterns at waves 2–4	Maltreatment was significantly associated with a blunted cortisol profile rather than a moderate or elevated profile at waves 2 and 3, but not wave 4 (moderate: Wave 2 OR = 0.44, *P <* .05; Wave 3 OR = 0.41, *P <* .01; Wave 4 OR = 0.87, *P =* ns; elevated: Wave 2 OR = 0.14, *P <* .01; Wave 3 OR = 0.31, *P <* .01; Wave 4 OR = 0.83, *P =* ns)
Rigterink (2010, USA) [42]	38 children aged 4–6 years, followed to age 9 years	Domestic violence by husband or wife in the previous 12 months, assessed at ages 5 and 9 years	Emotion regulation abilities at ages 5 and 9 years, measured via baseline vagal tone	Husband’s report of own and wife’s aggression (HDV) at age 5 years was associated with a significantly smaller increase in baseline vagal tone from the first to the second assessment, *P =* .01, even when controlling for HDV at age 9 years, *P =* .01; wife’s report of own and husband’s aggression at age 5 years was not significantly associated with alterations in baseline vagal tone, *P =* .57
Schmeer (2012, USA) [44]	Birth cohort from an urban area, followed to age 5 years (*N* = 1538)	Parental separation or divorce between ages 3 and 5 years	Change in overweight/obesity status between ages 3 and 5 years	Parental separation or divorce was associated with a significantly higher risk of becoming overweight/obese, RRR = 1.83 (SE = 0.55), *p* < .05
Shalev (2013, UK) [43]	118 pairs of 5-year-old identical twins, followed to age 12 years (*N* = 236); twin pairs with no violence exposure were matched on sex and SES status	Violence exposure (domestic violence, frequent bullying, and physical maltreatment) in one or both twins between ages 5 and 10 years	Telomere length at age 10 years	2 or more types of violence exposure were significantly associated with accelerated telomere erosion, *P =* .015, even when controlling for poor health and asthma, *P =* .028
Shenk (2015, USA) [47]	266 maltreated females aged 14-17 years and 128 nonmaltreated females (matched on age, race, family income, and single-parent household), followed for 4 years or until age 19 years	Substantiated maltreatment (physical abuse, sexual abuse, or physical neglect) within 12 months prior to recruitment	Obesity (BMI score >=30), assessed annually	Maltreatment was associated with a significantly increased risk for obesity, RR = 1.47 (1.03–2.08), *P =* .034
Wolf (2008, Canada) [39]	83 children aged 9–18 years, 50 with asthma and 33 medically healthy, followed for 6 months	Parental depressive symptoms during the past week, assessed at baseline	Interleukin-4 (IL-4) production at baseline and follow-up	Parental depressive symptoms were not associated with changes over time in IL-4 production, *P =* .19, in either children with asthma or healthy children
		Parental depressive symptoms during the past week, assessed at baseline	Eosinophil cationic protein concentrations (ECP) at baseline and follow-up	Parental depressive symptoms were associated with increases in children's ECP levels over time, *P =* .046, in both children with asthma and healthy children
Wolke (2014, UK) [58]	Birth cohort, followed to age 12 years (*N* = 6796)	Repeated or frequent bullying victimization in the past 6 months, assessed at age 8 years, age 10 years, or both	Parasomnias at age 12 years (nightmares, night terrors, and sleepwalking)	Bullying victimization at age 8 years was associated with a significant increase in risk of nightmares, OR = 1.23 (1.05–1.44), night terrors, OR = 1.39 (1.10–1.75), and any parasomnias, OR = 1.28 (1.11–1.47), but not sleepwalking, OR = 1.22 (0.99–1.50); bullying victimization at age 10 years was associated with a significant increase in risk of nightmares, OR = 1.62 (1.35–1.94), night terrors, OR = 1.53 (1.18–1.98), sleepwalking, OR = 1.40 (1.11–1.76), and any parasomnias, OR = 1.75 (1.48–2.07); bullying victimization at both ages 8 and 10 years was associated with a significant increase in risk of nightmares, OR = 1.82 (1.46–2.27), night terrors, OR = 2.01 (1.48–2.74), sleepwalking, OR = 1.71 (1.31–2.25), and any type of parasomnia, OR = 2.10 (1.72–2.58)
Wyman (2007, USA) [37]	158 children aged 5–10 years, followed for 18 months	Parental psychiatric symptoms (including depression and anxiety), ascertained at 4 assessments across 18 months	Number of illnesses and febrile illnesses in the 1 year following the second assessment	Parental psychiatric symptoms were associated with a significantly increased rate of illness, RR = 1.49 (1.12–1.97), *P =* .01, but not febrile illness, RR = 1.60 (0.94–2.73), *P =* .08
			Natural killer cell function at the fourth assessment and at all 4 assessments	Parental psychiatric symptoms were not significantly associated with increased natural killer cell function at the fourth assessment, *P =* .13, but were significantly associated with higher natural killer cell function at all 4 assessments, *P =* .01

Studies were prospective cohort unless otherwise noted. *95% CI* 95% confidence interval, *BMI* body mass index, *HR* hazard ratio, *PTSD* posttraumatic stress disorder, *OR* odds ratio, *RR* risk ratio, *RRR* relative risk ratio, *SES* socioeconomic status

^a^The nonexposed comparison represents the absence of the examined exposure

^b^Retrospective cohort study

### Bias assessment

The first two authors (D.O. and P.J.) independently assessed the risk of selection bias, information bias, and confounding for each study using the following 6 questions adapted from Busse and Guyatt’s Tool to Assess Risk of Bias in Cohort Studies [23]: 1) Was selection of exposed and nonexposed participants drawn from similar populations, 2) Was the follow-up adequate (e.g. percentage of attrition, percentage of missing data and type of missing data), 3) Can we be confident in the assessment of the exposure(s), 4) Can we be confident in the assessment of the outcome(s), 5) Did the study measure and adjust for potential confounding variables and covariates, 6) Can we be confident in the assessment of the confounding variables and covariates? The rating scale ranged from definitely yes (i.e. low risk of bias) to definitely no (i.e. high risk of bias). Rating disagreements between the 2 authors were resolved through discussion. As part of the assessment of potential confounding we also separately considered whether co-interventions were similar between the groups (i.e. social services).

## Results

After removal of duplicates, 15940 records were identified. Of these, 1179 studies investigated the relationship between childhood adversities and biological health outcomes, of which 380 (32%) reported outcomes in children. A majority of the studies with outcomes in children used a cross-sectional study design (241 studies, 63%). We identified 97 longitudinal cohort studies in children, and 24 met our final criteria (see flow diagram in Fig. 1 for the exclusion criteria). In addition, we identified 11 studies that met all inclusion criteria from the references section of the selected studies, for a total of 35 studies (Fig. 1).

### Bias assessment

**Fig. 2:**
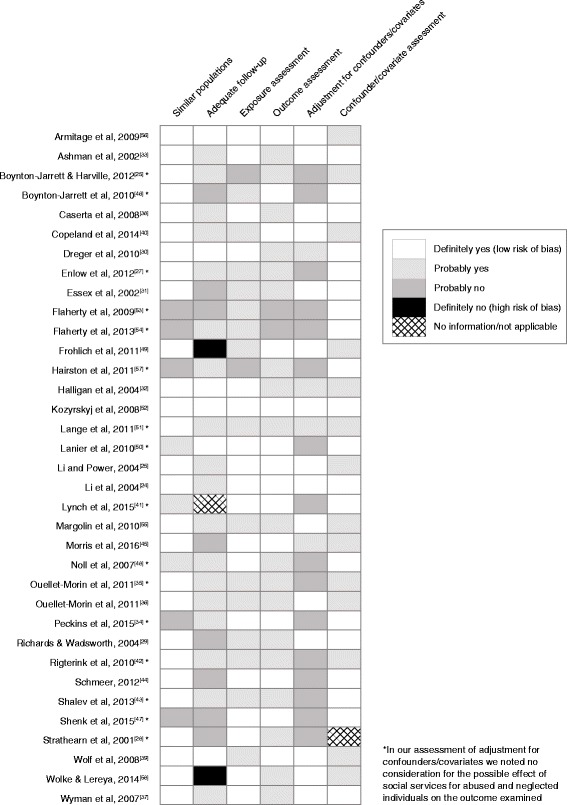
Risk of bias in selected studies

The bias assessment synthesis is displayed in Fig. 2. In 77% of the studies, exposed and unexposed participants were drawn from similar populations, suggesting a low risk of bias. In regard to adequate follow-up, 68% of the studies were assessed to have a low or moderately low risk of bias. The assessments of the exposure and the outcome were rated as having a low or moderately low risk of bias in 94% of the studies. In regard to confounders and covariates, 92% of the studies received a rating of low or moderately low risk of bias (Fig. 2). In addition, we observed that the majority of the studies examining maltreatment and intimate partner violence did not take into consideration the possible effect of social services for abused and neglected individuals on the outcome.

### Study characteristics

Of the 35 studies published between 2001 and 2015, 20 were published in 2010 and later. Thirty-three were prospective cohort studies and 2 were retrospective cohort studies. Half of the studies were conducted in the United States, and the remaining ones were conducted in 4 other countries. Sample size ranged from 18 to 13907 participants. Six studies examined developmental outcomes (Table 2) and 29 examined clinical outcomes (Table 3).

### Developmental outcomes

Six studies investigated developmental outcomes including physical, reproductive, and cognitive development. Studies found that adversity was associated with differences in height, age at menarche, and cognitive ability (Table 3).

#### Physical and reproductive development

Studies on physical development showed a weak association between parental divorce or separation and shorter height, but the association was not always statistically significant. Li et al found that for males whose parents divorced or separated, height was significantly lower at ages 7 and 11 years, but not at age 16 years. For females whose parents divorced or separated, height was lower on average at ages 7, 11, and 16 years, but not significantly lower [24]. Li and Power compared 2 generations of participants and found that cohort members whose parents divorced or separated were shorter on average, and offspring were taller on average, but these associations were not statistically significant [25]. Boynton-Jarrett and Harville examined children who were raised in a neglectful environment (e.g. physical neglect, bullying) and found that 1 form of neglectful environment (OR = 1.21, 95% CI: 1.03–1.43) and 2 or more forms (OR = 1.76, 95% CI: 1.41–2.20) were both significantly associated with late menarche [26].

#### Cognitive development

The reviewed studies reported an association between early adversity and significant delays in cognitive development. Enlow et al found that interpersonal trauma exposure during age 0–24 months, but not during age 24–64 months, was significantly associated with decreased cognitive scores at 24 to 96 months of age [27]. Similarly, Strathearn et al found a significant association between maltreatment and cognitive delay at age 4 years [28]. Richards and Wadsworth found that children who experienced parental death or divorce had lower cognitive ability scores at ages 8 and 15 years [29].

### Clinical outcomes

Of the 29 selected studies investigating clinical outcomes, 14 focused on biological markers. Of these studies, 7 examined markers of endocrine function (cortisol), 4 examined markers of inflammation and immune function, 2 examined markers of autonomic nervous function, and 1 examined telomere length. Other clinical outcomes reported included obesity (6 studies), asthma (3 studies), infections and illnesses (3 studies), somatic complaints (3 studies), and sleep (3 studies).

#### Biological markers of endocrine function

Studies on cortisol, a stress hormone with a diurnal pattern of secretion, give insight to the dysregulation of the stress response in children exposed to adversity. Of the 7 selected studies that examined cortisol, 4 investigated the effect of maternal mental health on cortisol secretion and showed that results varied with age of exposure. Dreger et al found that maternal postnatal and recurrent distress were significantly associated with higher midday serum cortisol levels in 7–10-year-olds [30]. Essex et al assessed children’s afternoon or evening salivary cortisol levels and found that those who were exposed to maternal depression both in infancy and at age 4.5 years had marginally significant, higher cortisol levels at age 4.5 years than did children never exposed or exposed only at age 4.5 years. In addition, children exposed to maternal depression only in infancy did not have significantly higher cortisol levels [31]. Halligan et al found that maternal postnatal depression was associated with higher, more variable morning salivary cortisol in 13-year-old children [32]. Ashman et al compared 7–8-year-old children of depressed and nondepressed mothers and found no effect of maternal depression on salivary cortisol levels or diurnal rhythm of cortisol [33].

In contrast, the 3 studies examining the effect of maltreatment on children’s cortisol production reported lower cortisol levels in maltreated children. Peckins et al found that maltreated children were more likely than nonmaltreated children to have a blunted cortisol profile rather than a moderate or an elevated profile, but by late adolescence there was no longer a difference [34]. Two studies on cortisol response to a psychosocial stress test in 12-year-old twins indicated that maltreated or frequently bullied children did not exhibit the expected increase in cortisol following the stress test [35, 36].

#### Biological markers of inflammation and immune function

The reviewed studies indicated alterations of immune and inflammatory response in children exposed to adversity. In a sample of children exposed to their parents’ psychiatric symptoms, Wyman et al [37] and Caserta et al [38] found that higher levels of symptoms were associated with enhanced natural killer cell response in children, suggesting that chronic stress may exert effects of cytotoxicity on the developing immune system. Wolf et al found that greater parental depressive symptoms at baseline predicted increases in children's profiles of asthma-relevant inflammatory markers (i.e. eosinophil cationic protein and interleukin-4), in both children with asthma and controls [39]. In 3 cohorts of children, Copeland et al did not find an association between bullying or teasing and C-reactive protein levels (a marker of inflammation in the body) [40].

#### Biological markers of autonomic nervous function

Lynch et al found that the interaction between exposure to neighborhood crime, exposure to maltreatment, and children’s genotype was associated with different patterns of respiratory sinus arrhythmia reactivity, a measure of the vagal tone indicating the physiological response of the autonomic nervous system to stress [41]. Rigterink et al found that exposure to domestic violence was associated with differences in the physiological regulation of emotion—measured as vagal tone—between exposed and nonexposed children, with smaller increases in baseline vagal tone in exposed children, suggesting a less adaptive development of regulatory functioning over time among these children [42].

#### Telomere length

One study investigated the relationship between violence exposure and telomere length, a marker of cellular age that also correlates with disease morbidity and mortality. Shalev et al observed stress-related accelerated telomere erosion in children who experienced 2 or more types of violence exposure, providing support for a mechanism linking cumulative childhood stress to potential lifelong and transgenerational health impacts [43].

#### Obesity

Evidence for early adversity influencing weight or body mass index (BMI) in childhood was mixed, with different types of adversity resulting in varying outcomes at different ages. Schmeer found that children whose mothers dissolved a union had an 80% higher risk of becoming overweight or obese between ages 3 and 5 years, as compared with children of stable married mothers [44]. Morris et al found that children whose parents had separated had a BMI 1.1% (95% CI: 0.2–2.0) higher at age 4 years in comparison with children whose parents remained together, but this diminished to 0.5% (95% CI: -1.3–2.2) by age 17 years [45]. Boynton-Jarrett et al found that children whose mothers reported chronic intimate partner violence (IPV) were more likely to be obese at age 5 years than were children whose mothers reported no IPV (OR = 1.80, 95% CI: 1.24–2.61), whereas children who were exposed to either early (up to age 12 months) or late (at age 3 and/or 5 years) maternal IPV did not have a significant increase in risk. Analyses stratified by sex indicated an increased risk for obesity only among females exposed to maternal chronic IPV (OR = 2.21, 95% CI: 1.30–3.75), as compared with males (OR = 1.66, 95% CI: 0.94–2.93) [46].

Studies on household dysfunction showed an effect on weight during early childhood, whereas studies on abuse and neglect reported an effect on weight during adolescence. Shenk et al found that maltreatment significantly increased the risk for obesity among female adolescents (RR = 1.47, 95% CI: 1.03–2.08, *P* = .034) [47]. Noll et al found a borderline, nonsignificant increase in risk for obesity during middle/late adolescence (age 15–19 years) for females who had been sexually abused (OR = 2.03, 95% CI: 0.54–4.60, *P* = .09); the relationship was not significant during childhood/early adolescence (age 6–14 years) [48].

Childhood adversity also showed an effect on children’s ability to manage their weight. Frohlich et al examined weight change among overweight and obese 7–15-year-olds participating in a weight reduction intervention. They found that psychosocial variables**—**family adversity, maternal depression, and maternal attachment style**—**were significantly predictive of long-term success in weight reduction [49].

#### Asthma

Studies reported that children exposed to childhood adversity had an increased risk for asthma. Lanier et al found that maltreatment significantly increased the risk for first hospital treatment for asthma (HR = 1.73, 95% CI: 1.47–2.04, *P* < .001) [50]. Lange et al found that paternal posttraumatic stress disorder (PTSD; OR = 1.08, 95% CI: 1.03–1.14, *P* = .003), paternal major depressive episode (OR = 9.95, 95% CI: 1.38–71.59, *P* = .02), and paternal antisocial behavior (OR = 1.09, 95% CI: 1.04–1.15, *P* < .001), as well as maternal depressive symptoms (OR = 1.13, 95% CI: 1.02–1.25, *P* = .02) and parental depression (OR = 1.70, 95% CI: 1.14–2.53, *P* = .01) were associated with recent asthma symptoms at age 1 year. At age 3 years, maternal depressive symptoms showed a significant association with asthma diagnosis (OR = 1.13, 95% CI: 1.01–1.27, *P* = .03) and a borderline, significant association with hospitalizations for asthma (OR = 1.16, 95% CI: 1.00–1.36, *P* = .05). Paternal major depressive episode and parental depression had a borderline, nonsignificant association with oral steroid treatment (OR = 3.03, 95% CI: 0.84–10.97, *P* = .09) and hospitalizations for asthma (OR = 1.86, 95% CI: 0.98–3.56, *P* = .06) [51]. In addition, Kozyrskyj et al found that maternal long-term distress was significantly associated with an increased risk of asthma at age 7 years (OR = 1.25, 95% CI: 1.01–1.55), but postpartum distress and short-term distress were not [52].

#### Infections and illnesses

An increased risk for infection was also reported to be associated with childhood adversity. Lanier et al found that maltreatment increased the risk for first hospital treatment for non-asthma-related cardiorespiratory disease (e.g. acute respiratory infection; HR = 2.07, 95% CI: 1.87–2.29, P < .001) as well as other infections (e.g. mycoses; HR = 2.09, 95% CI: 1.85-2.36, *P* < .001) [50]. Wyman et al studied a sample of children 5–10 years of age and found that after 18 months of follow-up, children of parents reporting higher levels of psychiatric symptoms had more illnesses (RR = 1.49, 95% CI: 1.12–1.97, *P* = .01), but not more febrile illnesses (RR = 1.60, 95% CI: 0.94–2.73, *P* = .08) [37]. At the 3-year follow-up, Caserta et al found that children of parents reporting higher levels of psychiatric symptoms continued to experience more illnesses (RR = 1.40, 95% CI: 1.06–1.85, *P* = .02), but they also experienced more febrile illnesses (RR = 1.77, 95% CI: 1.00–3.13, *P* = .05) [38].

#### Somatic complaints

The relationship between childhood adversity and somatic complaints (i.e. common physical complaints of uncertain origin such as headaches and nausea) varied by number and types of adversities. Flaherty et al found that experiencing 5 or more adversities during childhood was not associated with child somatic complaints reported by the child at age 12 years, but was associated with child somatic complaints reported by the caregiver (OR = 4.26, 95% CI: 1.17–15.5, *P* = .03) [53]. In a subsequent study, Flaherty et al examined somatic concerns reported at age 14 years and found a graded relationship between exposure to adversity across all ages and child somatic concerns reported by the caregiver; ORs (95% CI) for somatic concerns were 4.19 (0.50–34.90), 8.91 (1.15–68.83), and 9.25 (1.25–68.23) for 1, 2, and ≥3 adversities, respectively [54]. Margolin et al found that parent-to-youth aggression was significantly associated with a slightly increased risk of experiencing somatic symptoms (RR = 1.03, 95% CI: 1.01–1.05, *P* < .05), whereas marital physical aggression and community violence were not (RR = 1.01, 95% CI: 0.99–1.03 and RR = 1.03, 95% CI: 0.99–1.08, respectively) [55].

#### Sleep

Childhood adversity was also shown to affect children’s sleep. In a study on the effect of maternal depression on infant sleep, Armitage et al found that average sleep time in 24 hours did not differ between children of depressed and nondepressed mothers at 2 or 24 weeks, but nocturnal sleep was 97 min longer among children of nondepressed mothers at both 2 and 24 weeks, and these children had fewer awakenings during the night than did children of depressed mothers [56]. In examining the effect of maternal PTSD on infant sleep, Hairston et al. found a marginally significant association for infant waking after sleep onset and a nonsignificant association for amount of time awake during the night [57]. Wolke and Lereya found that bullying victimization at both ages 8 and 10 years was associated with nightmares (OR = 1.82, 95% CI: 1.46–2.27), night terrors (OR = 2.01, 95% CI: 1.48–2.74), sleepwalking (OR = 1.71, 95% CI: 1.31–2.25), and any type of parasomnia (OR = 2.10, 95% CI: 1.72–2.58) at age 12 years [58].

## Discussion

To date, the majority of scientific inquiry on childhood adversity and biological health outcomes has focused on adult outcomes. Furthermore, most pediatric studies have been cross-sectional in nature, making it difficult to determine the temporal relationship between exposures to adversity and biological health outcomes. This systematic review synthesized the longitudinal evidence on childhood adversity and pediatric health outcomes to help provide insight into the link between early manifestations of a dysregulated stress response and biological health outcomes. Outcomes identified represent a range of conditions, which reflects the multiple systems impacted by a chronically dysregulated stress response in childhood. We observed great variability in the results of the longitudinal studies included in this review, suggesting that it is critical to consider both the nature of the adversity and the individual characteristics of the exposed child.

The evidence from the selected longitudinal studies on cognitive delays, asthma, infections, somatic complaints, and sleep disruptions revealed associations between childhood adversity and increased risk of these conditions; this evidence adds to that from other nonlongitudinal studies in children [59–68]. This review also revealed mixed results related to obesity and physical development, with variability by age, sex, and exposure type. The variable findings may be due to bias related to loss to follow-up; however, genetic and environmental factors also may have influenced the heterogeneous response to childhood adversity [69–74]. Though a minority of the studies in this review focused on biomarkers, the growth of research and technology in this domain will help elucidate the biological mechanisms underlying the relationship between childhood adversity and health outcomes. Studies on cortisol included in this review used different sources (e.g. saliva, blood) and times of measurement, making it difficult to compare results across studies. In addition, the diverse effects of adversity observed on cortisol secretion may have been due to participant group differences and loss to follow-up. Nonetheless, the selected studies pointed to both elevated and blunted cortisol profiles, which might be indicative of a spectrum of responses that ranges from an initial increased activation of the stress response to a depletion of cortisol secretion over time, both of which can be detrimental to a child’s developing body [75, 76]. The reviewed studies also reported altered profiles of markers of inflammatory, immune, and autonomic nervous system functions, and epigenetic factors, highlighting the complexity of the biological response to adversity. It should be noted that, in general, the effect sizes reported across the various studies were small, which could be an accurate reflection of the small differences adversity exerts on the childhood outcomes we captured in this review or it could be an indication of methodological issues such as measurement or confounding.

### Strengths and limitations

This systematic review’s strengths include a wide publication window and a variety of search terms to capture studies on adversity. Limitations include exclusion of non-full-text and nonjournal publications, publication bias, and use of only 3 databases. We also did not contact authors for additional data or clarification. Another limitation is that definitions used for childhood adversity in the literature vary, making it challenging to perform a search to identify all sources of adversity. Furthermore, although most of the studies in this review examined more than one childhood adversity and some examined cumulative adversity, we did not explicitly focus on polyvictimization in our search, selection processes, or synthesis. Moreover, because the goal of this review was to broadly investigate relationships between a wide variety of adversities and health outcomes, we did not examine timing or severity of each individual type of adversity. Polyvictimization, timing, and severity are important aspects of childhood adversity [18, 77], and future studies in these areas may give insight to the complex mechanisms underlying the dysregulation of the stress response.

## Conclusions

This systematic review adds to the growing evidence on the relationship between childhood adversity and children’s health. In particular, this review provides support for two important concepts:

1. Childhood adversity affects brain development and multiple body systems, and the physiologic manifestations are detectable in childhood. Although pediatricians can often recognize the behavioral signs associated with exposure to adversity [78], the evidence from this review suggests that pediatricians should also consider such exposure when evaluating the differential diagnosis of pediatric conditions such as developmental delay, asthma, somatic complaints, recurrent infections requiring hospitalization, and sleep disruption, which were found to be consistently associated with adversity in the selected studies. Although clinical diagnostic guidelines for detecting a toxic stress response in children do not yet exist, these conditions might provide part of the basis for the development of such guidelines.
2. The variability in children’s response to adversity suggests complex underlying mechanisms, including the timing and severity of adversity, the experience of cumulative adversity, and the presence of protective factors (i.e. resilience, genetic and epigenetic constitution, individual socioeconomic status) that mitigate or exacerbate the impact of the exposure. This variability poses a challenge for the development of uniform diagnostic guidelines [79, 80].

Screening for exposure to childhood adversity, protective factors, and impacts of a toxic stress response could be the first step in providing targeted support for children at risk and their caregivers. In addition to screening, the American Academy of Pediatrics and other child health experts have also recommended strategies such as integrating behavioral healthcare into the pediatric home, offering parental support, providing peer-based education, and identifying community resources to help enhance resilience and mitigate the downstream effects of childhood adversity [81–88].

This systematic review provides a foundation for future research on the longitudinal relationship between childhood adversity and biological health outcomes. Future studies should examine in detail the findings we observed, taking into consideration timing and severity of adversity, the experience of cumulative adversity, and the presence of protective factors. This may be especially critical for outcomes for which we found mixed results, such as with physical development, obesity, and cortisol secretion. In addition, more large longitudinal studies are needed to develop a greater understanding of the causal pathways from exposure to childhood adversity to disease risk, to help the pediatric community develop services and interventions, identify protective factors, and prevent long-term negative health outcomes.

## Abbreviations

ACE: Adverse childhood experience
CI: Confidence interval
HR: Hazard ratio
IPV: Intimate partner violence
OR: Odds ratio
RR: Risk ratio

## References

[1] Shonkoff JP. Leveraging the biology of adversity to address the roots of disparities in health and development. *Proc Natl Acad Sci USA*. 2012; 10.1073/pnas.1121259109. 10.1073/pnas.1121259109 PMC3477384 23045654

[2] Fox SE, Levitt P, Nelson CA. How the timing and quality of early experiences influence the development of brain architecture. *Child Dev*. 2010; 10.1111/j.1467-8624.2009.01380.x. 10.1111/j.1467-8624.2009.01380.x PMC2846084 20331653

[3] Meaney MJ. Epigenetics and the biological definition of gene x environment interactions. *Child Dev*. 2010; 10.1111/j.1467-8624.2009.01381.x. 10.1111/j.1467-8624.2009.01381.x 20331654

[4] Shonkoff JP, Boyce WT, McEwen BS. Neuroscience, molecular biology, and the childhood roots of health disparities: building a new framework for health promotion and disease prevention. *JAMA*. 2009; 10.1001/jama.2009.754. 10.1001/jama.2009.754 19491187

[5] National Scientific Council on the Developing Child. Excessive Stress Disrupts the Architecture of the Developing Brain: Working Paper 3. Updated edition, 2014. http://developingchild.harvard.edu/resources/wp3. Accessed 19 Dec, 2015.

[6] McEwen BS (1998). Stress, adaptation, and disease. Allostasis and allostatic load. Ann NY Acad Sci..

[7] Lupien SJ. Effects of stress throughout the lifespan on the brain, behaviour and cognition. *Nat Rev Neurosci*. 2009; 10.1038/nrn2639. 10.1038/nrn2639 19401723

[8] Bucci M, Marques SS, Oh D, Harris NB. Toxic stress in children and adolescents. *Advances in Pediatrics*. 2016; 10.1016/j.yapd.2016.04.002. 10.1016/j.yapd.2016.04.002 27426909

[9] Felitti VJ, Anda RF, Nordenberg D (1998). Relationship of childhood abuse and household dysfunction to many of the leading causes of death in adults. The Adverse Childhood Experiences (ACE) Study. Am J Prev Med..

[10] Gilbert LK, Breiding MJ, Merrick MT, et al. Childhood adversity and adult chronic disease: an update from ten states and the District of Columbia, 2010. *Am J Prev Med*. 2015; 10.1016/j.amepre.2014.09.006. 10.1016/j.amepre.2014.09.006 25300735

[11] Campbell JA, Walker RJ, Egede LE. Associations between adverse childhood experiences, high-risk behaviors, and morbidity in adulthood. *Am J Prev Med*. 2016; 10.1016/j.amepre.2015.07.022. 10.1016/j.amepre.2015.07.022 PMC4762720 26474668

[12] Kalmakis KA, Chandler GE. Health consequences of adverse childhood experiences: a systematic review. *J Am Assoc Nurse Pract*. 2015; 10.1002/2327-6924.12215. 10.1002/2327-6924.12215 25755161

[13] Williamson DF, Thompson TJ, Anda RF, Dietz WH, Body FV. weight and obesity in adults and self-reported abuse in childhood. Int J Obes Relat Metab Disord. 2002; 10.1038/sj.ijo.0802038. 10.1038/sj.ijo.0802038 12119573

[14] Brown DW, Anda RF, Tiemeier H, et al. Adverse childhood experiences and the risk of premature mortality. Am J Prev Med. 2009; 10.1016/j.amepre.2009.06.021. 10.1016/j.amepre.2009.06.021 19840693

[15] Brown DW, Anda RF, Felitti VJ, et al. Adverse childhood experiences are associated with the risk of lung cancer: a prospective cohort study. BMC Public Health. 2010; 10.1186/1471-2458-10-20. 10.1186/1471-2458-10-20 PMC2826284 20085623

[16] Hardt J, Rutter M (2004). Validity of adult retrospective reports of adverse childhood experiences: review of the evidence. J Child Psychol Psychiatry..

[17] Reuben A, Moffitt TE, Caspi A, et al. Lest we forget: comparing retrospective and prospective assessments of adverse childhood experiences in the prediction of adult health. *J Child Psychol Psychiatry*. 2016; 10.1111/jcpp.12621. 10.1111/jcpp.12621 PMC5234278 27647050

[18] Bright MA, Knapp C, Hinojosa MS, Alford S, Bonner B. The comorbidity of physical, mental, and developmental conditions associated with childhood adversity: a population based study. *Matern Child Health J*. 2016; 10.1007/s10995-015-1915-7. 10.1007/s10995-015-1915-7 26694043

[19] Stein RE, Hurlburt MS, Heneghan AM, et al. Chronic conditions among children investigated by child welfare: a national sample. *Pediatrics*. 2013; 10.1542/peds.%202012-1774. 10.1542/peds.2012-1774 PMC4074665 23420907

[20] Sills MR, Shetterly S. Xu S, Magid D, Kempe A. Association between parental depression and children’s health care use. *Pediatrics*. 2007; 10.1542/peds.2006-2399. 10.1542/peds.2006-2399 17403826

[21] Moher D, Liberati A, Tetzlaff J, Altman DG. The PRISMA Group. Preferred reporting items for systematic reviews and meta-analyses: the PRISMA statement. *PLoS Med*. 2009; 10.1371/journal.pmed1000097. PMC3090117 21603045

[22] Burke Harris N, Renschler T (2015). *Center for Youth Wellness ACE-Questionnaire (CYW ACE-Q Child, Teen, Teen SR)*. Version 7.

[23] Tool to Assess Risk of Bias in Cohort Studies. 2014. https://www.evidencepartners.com/wp-content/uploads/2014/02/Tool-to-Assess-Risk-of-Bias-in-Cohort-Studies.doc. Accessed 22 May 2017.

[24] Li L, Manor O, Power C (2004). Early environment and child-to-adult growth trajectories in the 1958 British birth cohort. Am J Clin Nutr..

[25] Li L, Power C. Influences on childhood height: comparing two generations in the 1958 British birth cohort. *Int J Epidemiol*. 2004; 10.1093/ije/dyh325. 10.1093/ije/dyh325 15358746

[26] Boynton-Jarrett R, Harville EW. A prospective study of childhood social hardships and age at menarche. *Ann Epidemiol*. 2012; 10.1016/j.annepidem.2012.08.005. 10.1016/j.annepidem.2012.08.005 PMC3469794 22959664

[27] Enlow MB, Egeland B, Blood EA, Wright RO, Wright RJ. Interpersonal trauma exposure and cognitive development in children to age 8 years: a longitudinal study. *J Epidemiol Community Health*. 2012; 10.1136/jech-2011-200727. 10.1136/jech-2011-200727 PMC3731065 22493459

[28] Strathearn L, Gray PH, O’Callaghan MJ, Wood DO (2001). Childhood neglect and cognitive development in extremely low birth weight infants: a prospective study. Pediatrics..

[29] Richards M, Wadsworth ME. Long term effects of early adversity on cognitive function. *Arch Dis Child*. 2004; 10.1136/adc.2003.032490. 10.1136/adc.2003.032490 PMC1719683 15383435

[30] Dreger LC, Kozyrskyj AL, HayGlass KT, Becker AB, MacNeil BJ. Lower cortisol levels in children with asthma exposed to recurrent maternal distress from birth. *J Allergy Clin Immunol*. 2010; 10.1016/j.jaci.2009.09.051. 10.1016/j.jaci.2009.09.051 19962747

[31] Essex MJ, Klein MH, Cho E, Kalin NH. Maternal stress beginning in infancy may sensitize children to later stress exposure: effects on cortisol and behavior. *Biol Psychiatry*. 2002; http://dx.doi.org/10.1016/S0006-3223(02)01553-6. 10.1016/s0006-3223(02)01553-6 12372649

[32] Halligan SL, Herbert J, Goodyer IM, Murray L. Exposure to postnatal depression predicts elevated cortisol in adolescent offspring. *Biol Psychiatry*. 2004; http://dx.doi.org/10.1016/j.biopsych.2003.09.013. 10.1016/j.biopsych.2003.09.013 14960290

[33] Ashman SB, Dawson G, Panagiotides H, Yamada E, Wilkinson CW. Stress hormone levels of children of depressed mothers. *Dev Psychopathol*. 2002; https://doi.org/10.1017/S0954579402002080. 10.1017/s0954579402002080 12030695

[34] Peckins MK, Susman EJ, Negriff S, Noll J, Cortisol TPK. profiles: A test for adaptive calibration of the stress response system in maltreated and nonmaltreated youth. *Dev Psychopathol*. 2015; 10.1017/S0954579415000875. 10.1017/S0954579415000875 PMC5353977 26535937

[35] Ouellet-Morin I, Danese A, Bowes L, et al. A discordant monozygotic twin design shows blunted cortisol reactivity among bullied children. *J Am Acad Child Adolesc Psychiatry*. 2011; 10.1016/j.jaac.2011.02.015. 10.1016/j.jaac.2011.02.015 PMC3743243 21621141

[36] Ouellet-Morin I, Odgers CL, Danese A, et al. Blunted cortisol responses to stress signal social and behavioral problems among maltreated/bullied 12-year-old children. *Biol Psychiatry*. 2011; 10.1016/j.biopsych.2011.06.017. 10.1016/j.biopsych.2011.06.017 PMC3816750 21839988

[37] Wyman PA, Moynihan J, Eberly S, et al. Association of family stress with natural killer cell activity and the frequency of illnesses in children. *Arch Pediatr Adolesc Med*. 2007; 10.1001/archpedi.161.3.228. 10.1001/archpedi.161.3.228 17339503

[38] Caserta MT, O’Connor TG, Wyman PA, et al. The Associations between psychosocial stress and the frequency of illness, and innate and adaptive immune function in children. *Brain Behav Immun*. 2008; 10.1016/j.bbi.2008.01.007. 10.1016/j.bbi.2008.01.007 PMC2516370 18308510

[39] Wolf JM, Miller GE, Chen E. Parent psychological states predict changes in inflammatory markers in children with asthma and healthy children. *Brain Behav Immun*. 2008; 10.1016/j.bbi.2007.10.016. 10.1016/j.bbi.2007.10.016 PMC2441912 18068332

[40] Copeland WE, Wolke D, Lereya ST, et al. Childhood bullying involvement predicts low-grade systemic inflammation into adulthood. *PNAS*. 2014; 10.1073/pnas.1323641111. 10.1073/pnas.1323641111 PMC4040559 24821813

[41] Lynch M, Manly JT, Cicchetti D. A multilevel prediction of physiological response to challenge: interactions among child maltreatment, neighborhood crime, endothelial nitric oxide synthase gene (eNOS), and GABA(A) receptor subunit alpha-6 gene (GABRA6). *Dev Psychopathol*. 2015; 10.1017/S0954579415000887. 10.1017/S0954579415000887 PMC4635509 26535938

[42] Rigterink T, Fainsilber KL, Hessler DM. Domestic violence and longitudinal associations with children’s physiological regulation abilities. *J Interpers Violence*. 2010; 10.1177/0886260509354589. 10.1177/0886260509354589 PMC2950612 20587477

[43] Shalev I, Moffitt TE, Sugden K. Exposure to violence during childhood is associated with telomere erosion from 5 to 10 years of age: a longitudinal study. *Mol Psychiatry*. 2013; 10.1038/mp.2012.32. 10.1038/mp.2012.32 PMC3616159 22525489

[44] Schmeer KK. Family structure and obesity in early childhood. *Soc Sci Res*. 2012; http://dx.doi.org/10.1016/j.ssresearch.2012.01.007. 10.1016/j.ssresearch.2012.01.007 23017853

[45] Morris TT, Northstone K, Howe LD. Examining the association between early life social adversity and BMI changes in childhood: a life course trajectory analysis. *Pediatr Obes*. 2016; 10.1111/ijpo.12063. 10.1111/ijpo.12063 PMC4767691 26305573

[46] Boynton-Jarrett R, Fargnoli J, Suglia SF, Zuckerman B, Wright RJ. Association between maternal intimate partner violence and incident obesity in preschool-aged children: results from the Fragile Families and Child Well-being Study. *Arch Pediatr Adolesc Med*. 2010; 10.1001/archpediatrics.2010.94. 10.1001/archpediatrics.2010.94 PMC4586060 20530304

[47] Shenk CE, Noll JG, Peugh JL, Griffin AM, Bensman HE. Contamination in the Prospective Study of Child Maltreatment and Female Adolescent Health. *J Pediatr Psychol*. 2016; 10.1093/jpepsy/jsv017. 10.1093/jpepsy/jsv017 PMC4710181 25797944

[48] Noll JG, Zeller MH, Trickett PK, Obesity PFW. risk for female victims of childhood sexual abuse: a prospective study. *Pediatrics*. 2007; 10.1542/peds.2006-3058. 10.1542/peds.2006-3058 17606550

[49] Frohlich G, Pott W, Albayrak Ö, Hebebrand J, Conditions P-PU. of long-term success in a lifestyle intervention for overweight and obese youths. *Pediatrics*. 2011; 10.1542/peds.2010-3395. 10.1542/peds.2010-3395 21911346

[50] Lanier P, Jonson-Reid M, Stahlschmidt MJ, Drake B, Constantino J. Child maltreatment and pediatric health outcomes: a longitudinal study of low-income children. *J Pediatr Psychol*. 2010; 10.1093/jpepsy/jsp086. 10.1093/jpepsy/jsp086 PMC2910939 19797405

[51] Lange NE, Bunyavanich S, Silberg JL, Canino G, Rosner BA, Celedón JC. Parental psychosocial stress and asthma morbidity in Puerto Rican twins. *J Allergy Clin Immunol*. 2011; 10.1016/j.jaci.2010.11.010. 10.1016/j.jaci.2010.11.010 PMC3057225 21194742

[52] Kozyrskyj AL, Mai XM, McGrath P, Hayglass KT, Becker AB, Macneil B. Continued exposure to maternal distress in early life is associated with an increased risk of childhood asthma. *Am J Respir Crit Care Med*. 2008; http://dx.doi.org/10.1164/rccm.200703-381OC. 10.1164/rccm.200703-381OC 17932381

[53] Flaherty EG, Thompson R, Litrownik AJ, et al. Adverse childhood exposures and reported child health at age 12. *Acad Pediatr*. 2009; http://dx.doi.org/10.1016/j.acap.2008.11.003. 10.1016/j.acap.2008.11.003 19450774

[54] Flaherty EG, Thompson R, Dubowitz H, et al. Adverse childhood experiences and child health in early adolescence. *JAMA Pediatr*. 2013; 10.1001/jamapediatrics.2013.22. 10.1001/jamapediatrics.2013.22 PMC3732117 23645114

[55] Margolin G, Vickerman KA, Oliver PH, Gordis EB. Violence exposure in multiple interpersonal domains: cumulative and differential effects. *J Adolesc Health*. 2010; 10.1016/j.jadohealth.2010.01.020. 10.1016/j.jadohealth.2010.01.020 PMC2907247 20638013

[56] Armitage R, Flynn H, Hoffmann R, Vazquez D, Lopez J, Marcus S (2009). Early developmental changes in sleep in infants: the impact of maternal depression. Sleep..

[57] Hairston IS, Waxler E, Seng JS, Fezzey AG, Rosenblum KL, Muzik M. The role of infant sleep in intergenerational transmission of trauma. *Sleep*. 2011; 10.5665/%20SLEEP.1282. 10.5665/SLEEP.1282 PMC3174839 21966069

[58] Wolke D, Lereya ST. Bullying and parasomnias: a longitudinal cohort study. *Pediatrics*. 2014; 10.1542/peds.2014-1295. 10.1542/peds.2014-1295 25201799

[59] Wise LA, Palmer JR, Rothman EF, Childhood RL. abuse and early menarche: findings from the black women's health study. *Am J Public Health*. 2009; 10.2105/AJPH.2008.149005. 10.2105/AJPH.2008.149005 PMC2881664 19443822

[60] Gilbert AL, Bauer NS, Carroll AE, Child DSM. exposure to parental violence and psychological distress associated with delayed milestones. *Pediatrics*. 2013; 10.1542/peds.2013-1020. 10.1542/peds.2013-1020 PMC3838530 24190682

[61] Yang S, Kramer MS. Paternal alcohol consumption, family transition and child development in a former Soviet country. *Int J Epidemiol*. 2012; 10.1093/ije/dys071. 10.1093/ije/dys071 22586132

[62] Subramanian SV, Ackerson LK, Subramanyam MA, Wright RJ. Domestic violence is associated with adult and childhood asthma prevalence in India. *Int J Epidemiol*. 2007; 10.1093/ije/dym007. 10.1093/ije/dym007 17329314

[63] Gupta RS, Zhang X, Springston EE, et al. The association between community crime and childhood asthma prevalence in Chicago. *Ann Allergy Asthma Immunol*. 2010; 10.1016/j.anai.2009.11.047. 10.1016/j.anai.2009.11.047 20408339

[64] O’Donnell M, Nassar N, Leonard H, et al. Rates and types of hospitalisations for children who have subsequent contact with the child protection system: a population based case-control study. *J Epidemiol Community Health*. 2010; 10.1136/jech.2009.093393. 10.1136/jech.2009.093393 19778908

[65] Voerman JS, Vogel I, de Waart F, et al. Bullying, abuse and family conflict as risk factors for chronic pain among Dutch adolescents. *Eur J Pain*. 2015; 10.1002/ejp.689. 10.1002/ejp.689 25752511

[66] Collin SM, Tilling K, Joinson C, et al. Maternal and childhood psychological factors predict chronic disabling fatigue at age 13 years. *J Adolesc Health*. 2015; 10.1016/j.jadohealth.2014.09.002. 10.1016/j.jadohealth.2014.09.002 25448612

[67] van Tilburg MA, Runyan DK, Zolotor AJ, et al. Unexplained gastrointestinal symptoms after abuse in a prospective study of children at risk for abuse and neglect. *Ann Fam Med*. 2010; 10.1370/afm.1053. 10.1370/afm.1053 PMC2834720 20212300

[68] Schmid G, Schreier A, Meyer R, Wolke D. Predictors of crying, feeding and sleeping problems: a prospective study. *Child Care Health Dev*. 2011; 10.1111/j.1365-2214.%202010.01201.x. 10.1111/j.1365-2214.2010.01201.x 21299592

[69] Plomin R, Owen MJ, McGuffin P. The genetic basis of complex human behaviors. *Science*. 1994; 10.1126/science.8209254. 10.1126/science.8209254 8209254

[70] Bouchard TJ Jr. Genes, environment, and personality. *Science*. 1994; 10.1126/science.8209250. 10.1126/science.8209250 8209250

[71] Charmandari E, Tsigos C, Chrousos G. Endocrinology of the stress response. *Annu Rev Physiol*. 2005; 10.1146/annurev.physiol.67.040403.120816. 10.1146/annurev.physiol.67.040403.120816 15709959

[72] Habib KE, Gold PW, Chrousos GP. Neuroendocrinology of stress. *Endocrinol Metab Clin North Am*. 2001; http://dx.doi.org/10.1016/S0889-8529(05)70208-5. 10.1016/s0889-8529(05)70208-5 11571937

[73] Chrousos GP, Gold PW. The concepts of stress and stress system disorders. Overview of physical and behavioral homeostasis. *JAMA*. 1992; 10.1001/jama.1992.%2003480090092034. 1538563

[74] Tsigos C, Chrousos GP. Hypothalamic-pituitary-adrenal axis, neuroendocrine factors and stress. *J Psychosom Res*. 2002; http://dx.doi.org/10.1016/S0022-3999(02)00429-4. 10.1016/s0022-3999(02)00429-4 12377295

[75] Elzinga BM, Roelofs K, Tollenaar MS, Bakvis P, van Pelt J, Diminished SP. cortisol responses to psychosocial stress associated with lifetime adverse events a study among healthy young subjects. *Psychoneuroendocrinology*. 2008; 10.1016/j.psyneuen.2007.11.004. 10.1016/j.psyneuen.2007.11.004 18096322

[76] Gunnar MR, Frenn K, Wewerka SS, Van Ryzin MJ. Moderate versus severe early life stress: associations with stress reactivity and regulation in 10-12-year-old children. *Psychoneuroendocrinology*. 2009; 10.1016/j.psyneuen.2008.08.013. 10.1016/j.psyneuen.2008.08.013 PMC2670489 18835102

[77] Bethell CD, Newacheck P, Hawes E, Halfon N. Adverse childhood experiences: assessing the impact on health and school engagement and the mitigating role of resilience. *Health Aff (Millwood)*. 2014; 10.1377/hlthaff.2014.0914. 10.1377/hlthaff.2014.0914 25489028

[78] Sege RD, American Academy A-JL. of Pediatrics Committee on Child Abuse and Neglect, Council on Foster Care, Adoption, and Kinship Care; American Academy of Child and Adolescent Psychiatry Committee on Child Maltreatment and Violence; National Center for Child Traumatic Stress. Clinical considerations related to the behavioral manifestations of child maltreatment. *Pediatrics*. 2017; 10.1542/peds.2017-0100.

[79] Boyce WT, Biological EBJ (2005). sensitivity to context: I. An evolutionary–developmental theory of the origins and functions of stress reactivity. Dev Psychopathol..

[80] Belsky J, Pluess M. Beyond diathesis stress: differential susceptibility to environmental influences. *Psychol Bull*. 2009; 10.1037/a0017376. 10.1037/a0017376 19883141

[81] Garner AS, Shonkoff JP, Siegel BS, et al. Early childhood adversity, toxic stress, and the role of the pediatrician: translating developmental science into lifelong health. *Pediatrics*. 2012; 10.1542/peds.2011-2662. 10.1542/peds.2011-2662 22201148

[82] Traub F, Boynton-Jarrett R. Modifiable resilience factors to childhood adversity for clinical pediatric practice. *Pediatrics*. 2017; 10.1542/peds.2016-2569. 10.1542/peds.2016-2569 28557726

[83] Asarnow JR, Rozenman M, Wiblin J, Zeltzer L. Integrated medical-behavioral care compared with usual primary care for child and adolescent behavioral health: a meta-analysis. *JAMA Pediatr*. 2015; 10.1001/jamapediatrics.2015.1141. 10.1001/jamapediatrics.2015.1141 26259143

[84] Martin A, Barajas RG, Brooks-Gunn J, Hale L. Parenting services may be an opportunity for improving bedtime routines among at-risk preschoolers. *Behav Sleep Med*. 2011; 10.1080/15402002.2011.606771. 10.1080/15402002.2011.606771 PMC3197814 22003977

[85] Bethell C, Gombojav N, Solloway M, Wissow L. Adverse childhood experiences, resilience and mindfulness-based approaches. common denominator issues for children with emotional, mental, or behavioral problems. *Child Adolesc Psychiatr Clin N Am*. 2016; 10.1016/j.chc.2015.12.001. 10.1016/j.chc.2015.12.001 PMC4863233 26980120

[86] Nurius PS, Green S, Logan-Greene P, Borja S. Life course pathways of adverse childhood experiences toward adult psychological well-being: A stress process analysis. *Child Abuse Negl*. 2015; 10.1016/j.chiabu.2015.03.008. 10.1016/j.chiabu.2015.03.008 PMC4470711 25846195

[87] Flynn AB, Fothergill KE, Wilcox HC, et al. Primary care interventions to prevent or treat traumatic stress in childhood: a systematic review. *Acad Pediatr*. 2015; 10.1016/j.acap.2015.06.012. 10.1016/j.acap.2015.06.012 PMC4578291 26344717

[88] Hornor G. Childhood trauma exposure and toxic stress: what the PNP needs to know. J *Pediatr Health Care*. 2015; 10.1016/j.pedhc.2014.09.006. 10.1016/j.pedhc.2014.09.006 25697767

